# Supplementary material for: Discovery and application of insertion-deletion (INDEL) polymorphisms for QTL mapping of early life-history traits in Atlantic salmon
Source: BMC Genomics. 2010 Mar 8;11:156. doi: 10.1186/1471-2164-11-156 (PMC2838853; doi:10.1186/1471-2164-11-156)
Supplement: Additional file 2 — Information on developed 76 locus single-run INDEL panel in Atlantic salmon. Information on fluorescence labeling, primer concentrations, PCR pooling and links to alignments, INDEL motifs and GENESCAN (Burge and Karlin 1997) predictions of genes/exons are available in html format. [file 1471-2164-11-156-S2.ZIP › Additionalfile2/table13028.html]

Contig Alignment

```
Base	A B C D E F G H I J K   
1	. . . . . . . G . . .    
2	. . . . . . . A . . .    
3	. . . . . . . T . . .    
4	. . . . . . . T . . .    
5	. . . . . . . A . . .    
6	. . . . . . . C . . .    
7	. . . . . . . C . . .    
8	. . . . . . . A . . .    
9	. . . . . . . T . . .    
10	. . . . . . . G . . .    
11	. . . . . . . A . . .    
12	. . . . . . . T . . .    
13	. . . . . . . G . . .    
14	. . . . . . . A . . .    
15	. . . . . . . A . . .    
16	. . . . . . . A . . .    
17	. . . . . . . C . . .    
18	. . . . . . . A . . .    
19	. . . . . . . C . . .    
20	. . . . . . . T . . .    
21	. . . . . . . T . . .    
22	. . . . . . . T . . .    
23	. . . . . . . A . . .    
24	. . . . . . . T . . .    
25	. . . . . . . T . . .    
26	. . . . . . . T . . .    
27	. . . . . . . A . . .    
28	. . . . . . . C . . .    
29	. . . . . . . T . . .    
30	. . . . . . . A . . .    
31	. . . . . . . T . . .    
32	. . . . . . . C . . .    
33	. . . . . . . T . . .    
34	C . . . . . C T C C .    
35	T . T T . T T T T T .    
36	C . C C . C C C C C .    
37	C . C C . C C C C C .    
38	G . G G . G G G G G .    
39	T . T T . T T T T T .    
40	A . A A . A A A A A .    
41	C . C C . C C C C C .    
42	C . C C . C C C C C .    
43	A . A A . A A A A A .    
44	A . A A . A A A A A .    
45	A . A A . A A A A A .    
46	C C C C . C C C C C .    
47	A A A A . A A A A A .    
48	T T T T . T T T T T .    
49	G G G G . G G G G G .    
50	T T T T . T T T T T .    
51	A A A A . A A A A A .    
52	A A A A . A A A A A .    
53	A A A A . A A A A A .    
54	A A A A . A A A A A .    
55	T T T T . T T T T T .    
56	A A A A . A A A A A .    
57	A A A A . A A A A A .    
58	A A A A . A A A A A .    
59	A A A A . A A A A A .    
60	C C C C . C C C C C .    
61	A A A A . A A A A A .    
62	C C C C . C C C C C .    
63	A A A A . A A A A A .    
64	G G G G . G G G G G .    
65	T T T T . T T T T T .    
66	A A A A . A A A A A .    
67	A A A A . A A A A A .    
68	A A A A . A A A A A .    
69	A A A A . A A A A A .    
70	A A A A . A A A A A .    
71	A A A A . A A A A A .    
72	A A A A . A A A A A .    
73	G G G G . G G G G G .    
74	G G G G . G G G G G .    
75	C C C C . C C C C C .    
76	C C C C . C C C C C .    
77	A A A A . A A A A A .    
78	T T T T . T T T T T .    
79	A A A A . A A A A A .    
80	C C C C . C C C C C .    
81	A A A A . A A A A A .    
82	G G G G . G G G G G .    
83	T T T T . T T T T T .    
84	A A A A . A A A A A .    
85	A A A A . A A A A A .    
86	A A A A . A A A A A .    
87	A A A A . A A A A A .    
88	T T T T . T T T T T .    
89	A A A A . A A A A A .    
90	A A A A . A A A A A .    
91	A A A A . A A A A A .    
92	G G G G . G G G G G .    
93	A A A A . A A A A A .    
94	A A A A . A A A A A .    
95	C C C C . C C C C C .    
96	A A A A . A A A A A .    
97	C C C C . C C C C C .    
98	A A A A . A A A A A .    
99	T T T T . T T T T T .    
100	T T T T . T T T T T T    
101	C C C C . C C C C C C    
102	A A A A . A A A A A A    
103	A A A A . A A A A A A    
104	A A A A . A A A A A A    
105	T T T T . T T T T T T    
106	A A A A . A A A A A A    
107	G G G G . G G G G G G    
108	G G G G . G G G G G G    
109	G G G G . G G G G G G    
110	A A A A . A A A A A A    
111	C C C C . C C C C C C    
112	A A A A . A A A A A A    
113	A A A A . A A A A A A    
114	C C C C . C C C C C C    
115	A A A A . A A A A A A    
116	A A A A . A A A A A A    
117	G G G G . G G G G G G    
118	C C C C . C C C C C C    
119	T T T T . T T T T T T    
120	C C C C . C C C C C C    
121	T T T T . T T T T T T    
122	G G G G . G G G G G G    
123	C C C C . C C C C C C    
124	T T T T . T T T T T T    
125	A A A A . A A A A A A    
126	A A A A . A A A A A A    
127	A A A A . A A A A A A    
128	T T T T . T T T T T T    
129	G G G G . G G G G G G    
130	T T T T . T T T T T T    
131	C C C C . C C C C C C    
132	T T T T . T T T T T T    
133	T T T T . T T T T T T    
134	A A A A . A A A A A A    
135	C C C C . C C C C C C    
136	T T T T . T T T T T T    
137	G G G G . G G G G G G    
138	A A A A . A A A A A A    
139	A A A A . A A A A A A    
140	A A A A . A A A A A A    
141	A A A A . A A A A A A    
142	T T T T . T T T T T T    
143	C C C C . C C C C C C    
144	T T T T . T T T T T T    
145	A A A A . A A A A A A    
146	A A A A . A A A A A A    
147	A A A A . A A A A A A    
148	T T T T . T T T T T T    
149	C C C C . C C C C C C    
150	A A A A . A A A A A A    
151	G G G G . G G G G G G    
152	C C C C . C C C C C C    
153	A A A A . A A A A A A    
154	T T T T . T T T T T T    
155	C C C C . C C C C C C    
156	T T T T . T T T T T T    
157	G G G G . G G G G G G    
158	T T T T . T T T T T T    
159	A A A A . A A A A A A    
160	A A A A . A A A A A A    
161	A A A A . A A A A A A    
162	A A A A . A A A A A A    
163	T T T T . T T T T T T    
164	G G G G . G G G G G G    
165	T T T T . T T T T T T    
166	T T T T . T T T T T T    
167	C C C C . C C C C C C    
168	T T T T . T T T T T T    
169	A A A A . A A A A A A    
170	T T T T . T T T T T T    
171	A A A A . A A A A A A    
172	A A A A . A A A A A A    
173	G G G G . G G G G G G    
174	G G G G . G G G G G G    
175	G T T T . T T T T T T    
176	A A A A . A A A A A A    
177	C C C C . C C C C C C    
178	A A A A . A A A A A A    
179	T T T T . T T T T T T    
180	G G G G . G G G G G G    
181	A A A A . A A A A A A    
182	A A A A . A A A A A A    
183	G G G G . G G G G G G    
184	G G G G . G G G G G G    
185	A A A A . A A A A A A    
186	A A A A . A A A A A A    
187	G G G G . G G G G G G    
188	T T T T . T T T T T T    
189	G G G G . G G G G G G    
190	A A A A . A A A A A A    
191	A A A A . A A A A A A    
192	G G G G . G G G G G G    
193	T T T T . T T T T T T    
194	G G G G . G G G G G G    
195	T T T T . T T T T T T    
196	G G G G . G G G G G G    
197	T T T T . T T T T T T    
198	G G G G . G G G G G G    
199	T T T T . T T T T T T    
200	G G G G . G G G G G G    
201	T T - - . - T - T T -  5	 indel  
202	G G - - . - G - G G -  5	 indel  
203	T T - - . - T - T T -  5	 indel  
204	G G - - . - G - G G -  5	 indel  
205	T T - - . - T - T T -  5	 indel  
206	G G - - . - G - G G -  5	 indel  
207	T T T T . T T T T T T    
208	G G G G . G G G G G G    
209	G G G G . G G G G G G    
210	T T T T . T T T T T T    
211	T T T T . T T T T T T    
212	T T T T . T T T T T T    
213	A A A A . A A T A A T    
214	G G G G . G G G G G G    
215	G G G G . G G G G G G    
216	G G G G . G G G G G G    
217	C C C C . C C C C C C    
218	T T T T . T T T T T T    
219	T T T T . T T T T T T    
220	G G G G . G G G G G G    
221	G G G G . G G G G G G    
222	C C C C . C C C C C C    
223	C C C C . C C C C C C    
224	A A A A . A A A A A A    
225	G G G G . G G G G G G    
226	G G G G . G G G G G G    
227	T T T T . T T T T T T    
228	T T T T . T T T T T T    
229	A A A A . A A A A A A    
230	G G G G . G G G G G G    
231	C C C C . C C C C C C    
232	T T T T . T T T T T T    
233	T T T T T T T T T T T    
234	T T T T T T T T T T T    
235	A A A A A A A A A A A    
236	A A A A A A A A A A A    
237	T T T T T T T T T T T    
238	C C C C C C C C C C C    
239	C C C C C C C C C C C    
240	T T T T T T T T T T T    
241	A A A A A A A A A A A    
242	G G G G G G G G G G G    
243	C C C C C C C C C C C    
244	C C C C C C C C C C C    
245	T T T T T T T T T T T    
246	G G G G G G G G G G G    
247	G G G G G G G G G G G    
248	T T T T T T T T T T T    
249	C C C C C C C C C C C    
250	C C C C C C C C C C C    
251	A A A A A A A A A A A    
252	C C C C C C C C C C C    
253	G G G G G G G G G G G    
254	G G G G G G G G G G G    
255	C C C C C C C C C C C    
256	A A A A A A A A A A A    
257	T T T T T T T T T T T    
258	C C C C C C C C C C C    
259	C C C C C C C C C C C    
260	A A A A A A A A A A A    
261	G G G G G G G G G G G    
262	A A A A A A A A A A A    
263	A A A A A A A A A A A    
264	G G G G G G G G G G G    
265	G G G G G G G G G G G    
266	T T T T T T T T T T T    
267	T T T T T T T T T T T    
268	C C C C C C C C C C C    
269	T T T T T T T T T T T    
270	T T T T T T T T T T T    
271	G G G G G G G G G G G    
272	G G G G G G G G G G G    
273	A A A A A A A A A A A    
274	G G G G G G G G G G G    
275	T T T T T T T T T T T    
276	C C C C C C C C C C C    
277	C C C C C C C C C C C    
278	A A A A A A A A A A A    
279	G G G G G G G G G G G    
280	G G G G G G G G G G G    
281	G G G G G G G G G G G    
282	C C C C C C C C C C C    
283	C C C C C C C C C C C    
284	A A A A A A A A A A A    
285	A A A A A A A A A A A    
286	G G G G G G G G G G G    
287	G G G G G G G G G G G    
288	T T T T T T T T T T T    
289	G G G G G G G G G G G    
290	T T T T T T T T T T T    
291	G G G G G G G G G G G    
292	G G G G G G G G G G G    
293	G G G G G G G G G G G    
294	G C C C C C C C C C C    
295	. G G G G G G G G G G    
296	. A A A A A A A A A A    
297	. C C C C C C C C C C    
298	. G G G G G G G G G G    
299	. C C C C C C C C C C    
300	. C C C C C C C C C C    
301	. C C C C C C C C C C    
302	. A A A A A A A A A A    
303	. A A A A A A A A A A    
304	. C C C C C C C C C C    
305	. A A A A A A A A A A    
306	. T T T T T T T T T T    
307	. C C C C C C C C C C    
308	. T T T T T T T T T T    
309	. G G G G G G G G G G    
310	. C C C C C C C C C C    
311	. C C C C C C C C C C    
312	. T T T T T T T T T T    
313	. C C C C C C C C C C    
314	. T T T T T T T T T T    
315	. G G G G G G G G G G    
316	. A A A A A A A A A A    
317	. C C C C C C C C C C    
318	. A A A A A A A A A A    
319	. C C C C C C C C C C    
320	. T T T T T T T T T T    
321	. C C C C C C C C C C    
322	. C C C C C C C C C C    
323	. C C C T C C C C C C    
324	. C C C C C C C C C C    
325	. C C C C C C C C C C    
326	. T T T T T T T T T T    
327	. T T T T T T T T T T    
328	. C C C C C C C C C C    
329	. A A A A A A A A A A    
330	. G G G G G G G G G G    
331	. T T T T T T T T T T    
332	. G G G G G G G G G G    
333	. A A A A A A A A A A    
334	. T T T T T T T T T T    
335	. G G G G G G G G G G    
336	. T T T T T T T T T T    
337	. C C C C C C C C C C    
338	. A A A A A A A A A A    
339	. C C C C C C C C C C    
340	. A A A A A A A A A A    
341	. G G G G G G G G G G    
342	. A A A A A A A A A A    
343	. G G G G G G G G G G    
344	. T T T T T T T T T T    
345	. T T T T T T T T T T    
346	. C C C C C C C C C C    
347	. T T T T T T T T T T    
348	. G G G G G G G G G G    
349	. C C C C C C C C C C    
350	. T T T T T T T T T T    
351	. G G G G G G G G G G    
352	. G G G G G G G G G G    
353	. A A A A A A A A A A    
354	. C C C C C C C C C C    
355	. C C C C C C C C C C    
356	. A A A A A A A A A A    
357	. A A A A A A A A A A    
358	. T T T T T T T T T T    
359	. C C C C C C C C C C    
360	. G G G G G G G G G G    
361	. C C C C C C C C C C    
362	. A A A A A A A A A A    
363	. T T T T T T T T T T    
364	. C C C C C C C C C C    
365	. T T T T T T T T T T    
366	. T T T T T T T T T T    
367	. A A A A A A A A A A    
368	. G G G G G G G G G G    
369	. C C C C C C C C C C    
370	. G G G G G G G G G G    
371	. A A A A A A A A A A    
372	. T T T T T T T T T T    
373	. T T T T T T T T T T    
374	. A A A A A A A A A A    
375	. A A A A A A A A A A    
376	. C C C C C C C C C C    
377	. T T T T T T T T T T    
378	. C C C C C C C C C C    
379	. T T T T T T T T T T    
380	. C C C C C C C C C C    
381	. C C C C C C C C C C    
382	. C C C C C C C C C C    
383	. A A A A A A A A A A    
384	. G G G G G G G G G G    
385	. A A A A A A A A A A    
386	. T T T T T T T T T T    
387	. C C C C C C C C C C    
388	. C C C C C C C C C C    
389	. T T T T T T T T T T    
390	. T T T T T T T T T T    
391	. G G G G G G G G G G    
392	. T T T T T T T T T T    
393	. T T T T T T T T T T    
394	. C C C C C C C C C C    
395	. A A A A A A A A A A    
396	. G G G G G G G G G G    
397	. T T T T T T T T T T    
398	. A A A A A A A A A A    
399	. G G G G G G G G G G    
400	. C C C C C C C C C C    
401	. C C C C C C C C C C    
402	. T T T T T T T T T T    
403	. C C C C C C C C C C    
404	. T T T T T T T T T T    
405	. C C C C C C C C C C    
406	. A A A A A A A A A A    
407	. G G G G G G G G G G    
408	. T T T T T T T T T T    
409	. T T T T T T T T T T    
410	. C C C C C C C C C C    
411	. C C C C C C C C C C    
412	. C C C C C C C C C C    
413	. T T T T T T T T T T    
414	. C C C C C C C C C C    
415	. G G G G G G G G G G    
416	. A A A A A A A A A A    
417	. T T T T T T T T T T    
418	. C C C C C C C C C C    
419	. T T T T T T T T T T    
420	. C C C C C C C C C C    
421	. T T T T T T T T T T    
422	. G G G G G G G G G G    
423	. T T T T T T T T T T    
424	. T T T T T T T T T T    
425	. C C C C C C C C C C    
426	. T T T T T T T T T T    
427	. G G G G G G G G G G    
428	. G G G G G G G G G G    
429	. C C C C C C C C C C    
430	. A A A A A A A A A A    
431	. G G G G G G G G G G    
432	. A A A A A A A A A A    
433	. G G G G G G G G G G    
434	. C C C C C C C C C C    
435	. T T T T T T T T T T    
436	. G G G G G G G G G G    
437	. T T T T T T T T T T    
438	. G G G G G G G G G G    
439	. T T T T T T T T T T    
440	. A A A A A A A A A A    
441	. G G G G G G G G G G    
442	. T T T T T T T T T T    
443	. G G G G G G G G G G    
444	. A A A A A A A A A A    
445	. T T T T T T T T T T    
446	. G G G G G G G G G G    
447	. G G G G G G G G G G    
448	. T T T T T T T T T T    
449	. A A A A A A A A A A    
450	. G G G G G G G G G G    
451	. A A A A A A A A A A    
452	. A A A A A A A A A A    
453	. C C C C C C C C C C    
454	. T T T T T T T T T T    
455	. T T T T T T T T T T    
456	. C C C C C C C C C C    
457	. A A A A A A A A A A    
458	. T T T T T T T T T T    
459	. C C C C C C C C C C    
460	. A A A A A A A A A A    
461	. A A A A A A A A A A    
462	. C C C C C C C C C C    
463	. A A A A A A A A A A    
464	. C C C C C C C C C C    
465	. T T T T T T T T T T    
466	. A A A A A A A A A A    
467	. C C C C C C C C C C    
468	. G G G G G G G G G G    
469	. G G G G G G G G G G    
470	. A A A A A A A A A A    
471	. T T T T T T T T T T    
472	. C C C C C C C C C C    
473	. A A A A A A A A A A    
474	. G G G G G G G G G G    
475	. A A A A A A A A A A    
476	. C C C C C C C C C C    
477	. T T T T T T T T T T    
478	. A A A A A A A A A A    
479	. C C C C C C C C C C    
480	. G G G G G G G G G G    
481	. A A A A A A A A A A    
482	. A A A A A A A A A A    
483	. G G G G G G G G G G    
484	. A A A A A A A A A A    
485	. G G G G G G G G G G    
486	. T T T T T T T T T T    
487	. T T T T T T T T T T    
488	. A A A A A A A A A A    
489	. A A A A A A A A A A    
490	. C C C C C C C C C C    
491	. C C C C C C C C C C    
492	. C C C C C C C C C C    
493	. C C C C C C C C C C    
494	. A A A A A A A A A A    
495	. C C C C C C C C C C    
496	. G G G G G G G G G G    
497	. G G G G G G G G G G    
498	. C C C C C C C C C C    
499	. C C C C C C C C C C    
500	. T T T T T T T T T T    
501	. T T T T T T T T T T    
502	. G G G G G G G G G G    
503	. A A A A A A A A A A    
504	. C C C C C C C C C C    
505	. A A A A A A A A A A    
506	. G G G G G G G G G G    
507	. C C C C C C C C C C    
508	. A A A A A A A A A A    
509	. T T T T T T T T T T    
510	. T T T T T T T T T T    
511	. T T T T T T T T T T    
512	. G G G G G G G G G G    
513	. G G G G G G G G G G    
514	. G G G G G G G G G G    
515	. T T T T T T T T T T    
516	. A A A A A A A A A A    
517	. T T T T T T T T T T    
518	. T T T T T T T T T T    
519	. C C C C C C C C C C    
520	. T T T T T T T T T T    
521	. G G G G G G G G G G    
522	. A A A A A A A A A A    
523	. G G G G G G G G G G    
524	. G G G G G G G G G G    
525	. G G G G G G G G G G    
526	. G G G G G G G G G G    
527	. T T T T T T T T T T    
528	. G G G G G G G G G G    
529	. C C C C C C C C C C    
530	. A A A A A A A A A A    
531	. G G G G G G G G G G    
532	. T T T T T T T T T T    
533	. G G G G G G G G G G    
534	. T T T T T T T T T T    
535	. G G G G G G G G G G    
536	. T T T T T T T T T T    
537	. T T T T T T T T T T    
538	. G G G G G G G G G G    
539	. A A A A A A A A A A    
540	. C C C C C C C C C C    
541	. A A A A A A A A A A    
542	. T T T T T T T T T T    
543	. C C C C C C C C C C    
544	. A A A A A A A A A A    
545	. T T T T T T T T T T    
546	. T T T T T T T T T T    
547	. C C C C C C C C C C    
548	. T T T T T T T T T T    
549	. T T T T T T T T T T    
550	. C C C C C C C C C C    
551	. A A A A A A A A A A    
552	. G G G G G G G G G G    
553	. T T T T T T T T T T    
554	. T T T T T T T T T T    
555	. G G G G G G G G G G    
556	. C C C C C C C C C C    
557	. A A A A A A A A A A    
558	. C C C C C C C C C C    
559	. C C C C C C C C C C    
560	. A A A A A A A A A A    
561	. C C C C C C C C C C    
562	. A A A A A A A A A A    
563	. T T T T T T T T T T    
564	. A C C C C C C C C C    
565	. . T T T T T T T T T    
566	. . C C C C C C C C C    
567	. . T T T T T T T T T    
568	. . C C C C C C C C C    
569	. . A A A A A A A A A    
570	. . C C C C C C C C C    
571	. . C C C C C C C C C    
572	. . A A A A A A A A A    
573	. . T T T T T T T T T    
574	. . C C C C C C C C C    
575	. . G G G G G G G G G    
576	. . C C C C C C C C C    
577	. . C C C C C C C C C    
578	. . A A A A A A A A A    
579	. . T T T T T T T T T    
580	. . G G G G G G G G G    
581	. . A A A A A A A A A    
582	. . C C C C C C C C C    
583	. . A A A A A A A A A    
584	. . C C C C C C C C C    
585	. . C C C C C C C C C    
586	. . C C C C C C C C C    
587	. . A A A A A A A A A    
588	. . C C C C C C C C C    
589	. . A A A A A A A A A    
590	. . T T T T T T T T T    
591	. . A A A A A A A A A    
592	. . C C C C C C C C C    
593	. . A A A A A A A A A    
594	. . C C C C C C C C C    
595	. . C C C C C C C C C    
596	. . T T T T T T T T T    
597	. . G G G G G G G G G    
598	. . G G G G G G G G G    
599	. . T T T T T T T T T    
600	. . C C C C C C C C C    
601	. . A A A A A A A A A    
602	. . T T T T T T T T T    
603	. . C C C C C C C C C    
604	. . T T T T T T T T T    
605	. . G G G G G G G G G    
606	. . T T T T T T T T T    
607	. . C C C C C C C C C    
608	. . C C C C C C C C C    
609	. . T T T T T T T T T    
610	. . G G G G G G G G G    
611	. . T T T T T T T T T    
612	. . C C C C C C C C C    
613	. . G G G G G G G G G    
614	. . A A A A A A A A A    
615	. . G G G G G G G G G    
616	. . C C C C C C C C C    
617	. . T T T T T T T T T    
618	. . C C C C C C C C C    
619	. . T T T T T T T T T    
620	. . G G G G G G G G G    
621	. . C C C C C C C C C    
622	. . C C C C C C C C C    
623	. . G G G G G G G G G    
624	. . T T T T T T T T T    
625	. . G G G G G G G G G    
626	. . G G G G G G G G G    
627	. . C C C C C C C C C    
628	. . T T T T T T T T T    
629	. . G G G G G G G G G    
630	. . T T T T T T T T T    
631	. . A A A A A A A A A    
632	. . . G G G G G G G G    
633	. . . G G G G G G G G    
634	. . . C C C C C C C C    
635	. . . T T T T T T T T    
636	. . . G G G G G G G G    
637	. . . T T T T T T T T    
638	. . . G G G G G G G G    
639	. . . G G G G G G G G    
640	. . . A A A A A A A A    
641	. . . G G G G G G G G    
642	. . . C C C C C C C C    
643	. . . T T T T T T T T    
644	. . . G G G G G G G G    
645	. . . A A A A A A A A    
646	. . . G G G G G G G G    
647	. . . G G G G G G G G    
648	. . . C C C C C C C C    
649	. . . C C C C C C C C    
650	. . . T T T T T T T T    
651	. . . T T T T T T T T    
652	. . . T T T T T T T T    
653	. . . T G G G G G G G    
654	. . . . G G G G G G G    
655	. . . . T T T T T T T    
656	. . . . G G G G G G G    
657	. . . . G G G G G G G    
658	. . . . T T T T T T T    
659	. . . . T T T T T T T    
660	. . . . T T T T T T T    
661	. . . . G G G G G G G    
662	. . . . T T T T T T T    
663	. . . . C C C C C C C    
664	. . . . T T T T T T T    
665	. . . . G G G G G G G    
666	. . . . G G G G G G G    
667	. . . . A A A A A A A    
668	. . . . G G G G G G G    
669	. . . . G G G G G G G    
670	. . . . A A A A A A A    
671	. . . . G G G G G G G    
672	. . . . G G G G G G G    
673	. . . . T T T T T T T    
674	. . . . G G G G G G G    
675	. . . . T T T T T T T    
676	. . . . G G G G G G G    
677	. . . . G G G G G G G    
678	. . . . C C C C C C C    
679	. . . . T T T T T T T    
680	. . . . T T T T T T T    
681	. . . . G G G G G G G    
682	. . . . G G G G G G G    
683	. . . . T T T T T T T    
684	. . . . C C C C C C C    
685	. . . . T T T T T T T    
686	. . . . G G G G G G G    
687	. . . . C C C C C C C    
688	. . . . . T T T T T T    
689	. . . . . G G G G G G    
690	. . . . . A A A A A A    
691	. . . . . C C C C C C    
692	. . . . . T T T T T T    
693	. . . . . C C C C C C    
694	. . . . . C C C C C C    
695	. . . . . T T T T T T    
696	. . . . . C C C C C C    
697	. . . . . C C C C C C    
698	. . . . . T T T T T T    
699	. . . . . C C C C C C    
700	. . . . . A A A A A A    
701	. . . . . T T T T T T    
702	. . . . . G G G G G G    
703	. . . . . C C C C C C    
704	. . . . . G G G G G G    
705	. . . . . A A A A A A    
706	. . . . . A A A A A A    
707	. . . . . G G G G G G    
708	. . . . . C C C C C C    
709	. . . . . T T T T T T    
710	. . . . . G G G G G G    
711	. . . . . T T T T T T    
712	. . . . . T T T T T T    
713	. . . . . C C C C C C    
714	. . . . . T T T T T T    
715	. . . . . T G G G G G    
716	. . . . . . A A A A A    
717	. . . . . . A A A A A    
718	. . . . . . C C C C C    
719	. . . . . . T T T T T    
720	. . . . . . C C C C C    
721	. . . . . . C C C C C    
722	. . . . . . T T T T T    
723	. . . . . . G G G G G    
724	. . . . . . T T T T T    
725	. . . . . . T T T T T    
726	. . . . . . G G G G G    
727	. . . . . . A A A A A    
728	. . . . . . A A A A A    
729	. . . . . . C C C C C    
730	. . . . . . T T T T T    
731	. . . . . . T T T T T    
732	. . . . . . G G G G G    
733	. . . . . . C C C C C    
734	. . . . . . A A A A A    
735	. . . . . . C C C C C    
736	. . . . . . A A A A A    
737	. . . . . . G G G G G    
738	. . . . . . G G G G G    
739	. . . . . . A A A A A    
740	. . . . . . T T T T T    
741	. . . . . . C C C C C    
742	. . . . . . C C C C C    
743	. . . . . . A A A A A    
744	. . . . . . G G G G G    
745	. . . . . . G G G G G    
746	. . . . . . T T T T T    
747	. . . . . . G G G G G    
748	. . . . . . T T T T T    
749	. . . . . . T T T T T    
750	. . . . . . T T T T T    
751	. . . . . . C C C C C    
752	. . . . . . . T T T T    
753	. . . . . . . C C C C    
754	. . . . . . . C C C C    
755	. . . . . . . T T T T    
756	. . . . . . . C C C C    
757	. . . . . . . C C C C    
758	. . . . . . . T T T T    
759	. . . . . . . G G G G    
760	. . . . . . . C C C C    
761	. . . . . . . T T T T    
762	. . . . . . . C C C C    
763	. . . . . . . C C C C    
764	. . . . . . . A A A A    
765	. . . . . . . G G G G    
766	. . . . . . . C C C C    
767	. . . . . . . C C C C    
768	. . . . . . . A A A A    
769	. . . . . . . C C C C    
770	. . . . . . . C C C C    
771	. . . . . . . G G G G    
772	. . . . . . . G G G G    
773	. . . . . . . C C C C    
774	. . . . . . . T T T T    
775	. . . . . . . G G G G    
776	. . . . . . . T T T T    
777	. . . . . . . C C C C    
778	. . . . . . . T T T T    
779	. . . . . . . G G G G    
780	. . . . . . . T T T T    
781	. . . . . . . C C C C    
782	. . . . . . . A A A A    
783	. . . . . . . G G G G    
784	. . . . . . . C C C C    
785	. . . . . . . A A A A    
786	. . . . . . . T T T T    
787	. . . . . . . G G G G    
788	. . . . . . . T T T T    
789	. . . . . . . C C C C    
790	. . . . . . . C C C C    
791	. . . . . . . T . . T    
792	. . . . . . . C . . C    
793	. . . . . . . T . . T    
794	. . . . . . . C . . C    
795	. . . . . . . T . . T    
796	. . . . . . . T . . T    
797	. . . . . . . T . . T    
798	. . . . . . . G . . G    
799	. . . . . . . T . . T    
800	. . . . . . . C . . C    
801	. . . . . . . T . . T    
802	. . . . . . . A . . A    
803	. . . . . . . C . . C    
804	. . . . . . . G . . G    
805	. . . . . . . G . . G    
806	. . . . . . . A . . A    
807	. . . . . . . . . . G    
808	. . . . . . . . . . A    
809	. . . . . . . . . . G    
810	. . . . . . . . . . T    
811	. . . . . . . . . . A    
812	. . . . . . . . . . T    
813	. . . . . . . . . . C    
814	. . . . . . . . . . C    
815	. . . . . . . . . . T    
816	. . . . . . . . . . C    
817	. . . . . . . . . . C    
818	. . . . . . . . . . A    
819	. . . . . . . . . . C    
820	. . . . . . . . . . C    
821	. . . . . . . . . . C    
822	. . . . . . . . . . T    
823	. . . . . . . . . . C    
824	. . . . . . . . . . T    
825	. . . . . . . . . . C    
826	. . . . . . . . . . C    
827	. . . . . . . . . . C    
828	. . . . . . . . . . T    
829	. . . . . . . . . . C    
830	. . . . . . . . . . T    
831	. . . . . . . . . . C    
832	. . . . . . . . . . C    
833	. . . . . . . . . . C    
834	. . . . . . . . . . A    
835	. . . . . . . . . . G    
836	. . . . . . . . . . A    
837	. . . . . . . . . . C    
838	. . . . . . . . . . A    
839	. . . . . . . . . . G    
840	. . . . . . . . . . A    
841	. . . . . . . . . . C    
842	. . . . . . . . . . C    
843	. . . . . . . . . . T    
844	. . . . . . . . . . C    
845	. . . . . . . . . . G    
846	. . . . . . . . . . C    
847	. . . . . . . . . . A    
848	. . . . . . . . . . T    
849	. . . . . . . . . . C    
850	. . . . . . . . . . C    
851	. . . . . . . . . . T    
852	. . . . . . . . . . C    
853	. . . . . . . . . . C    
854	. . . . . . . . . . A    
855	. . . . . . . . . . C    
856	. . . . . . . . . . C    
857	. . . . . . . . . . C    
858	. . . . . . . . . . C    
859	. . . . . . . . . . C    
860	. . . . . . . . . . C    
861	. . . . . . . . . . C
```
